# Zinc finger protein 91 accelerates tumour progression by activating β‐catenin signalling in pancreatic cancer

**DOI:** 10.1111/cpr.13031

**Published:** 2021-03-23

**Authors:** Neng Tang, Shanshan Xu, Taiyu Song, Yudong Qiu, Jian He, Xiao Fu

**Affiliations:** ^1^ Nanjing Drum Tower Hospital The Affiliated Hospital of Nanjing University Medical School Nanjing China

**Keywords:** bioinformatics, chemoresistance, pancreatic cancer, tumour biology, zinc finger protein 91, β‐catenin

## Abstract

**Objectives:**

ZFP91, an E3 ligase, has been reported to possess cancer‐promoting functions. This study aimed to elucidate the exact role of ZFP91 in tumour progression of pancreatic cancer and underlying mechanisms.

**Materials and Methods:**

We analysed the correlation between ZFP91 expression and pancreatic cancer through TCGA and GEO data sets. Growth curve, colony formation, wound healing and transwell invasion assays were conducted to evaluate proliferation, migration and invasion of lentivirus transfected pancreatic cancer cells. GSEA and Western blot analysis were performed to validate the regulatory effect of ZFP91 on β‐catenin. Drug response curve and orthotopic implantation model reflected the sensitivity of chemotherapies.

**Results:**

ZFP91 overexpression is prevalent in pancreatic cancer and negatively correlated with overall survival. ZFP91 knock‐down attenuated proliferation, migration and invasion of pancreatic cancer cells. β‐catenin was a downstream gene of ZFP91, and its agonist could reverse the phenotype. ZFP91 promoted EMT and chemoresistance in pancreatic cancer.

**Conclusions:**

We demonstrated that ZFP91 promoted pancreatic cancer proliferation, migration and invasion through activating β‐catenin signalling. EMT and chemoresistance were also regulated by ZFP91. ZFP91 might be a potential therapeutic target for pancreatic cancer.

## Graphical abstract

ZFP91 promoted proliferation, migration and invasion in pancreatic cancer through the activation of β‐catenin signalling pathway, which was conductive to tumour progression. ZFP91 also facilitated EMT and chemoresistance in pancreatic cancer. ZFP91 might be a promising therapeutic target of pancreatic cancer.

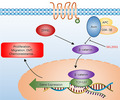

## 1 INTRODUCTION

Pancreatic cancer is a lethal disease with a 5‐year survival less than 9%.^1^ The diagnosis of pancreatic cancer is often at late stage and over 80% of patients with pancreatic cancer are not suitable for surgical resection.^2^ Chemotherapy and targeted therapy are now the most widely used treatments for pancreatic cancer. However, due to the rapidly developed resistance to drugs, the survival of patients is far from satisfactory.^3^ Therefore, it is imperative to uncover the underlying mechanisms responsible for tumour progression in pancreatic cancer, so as to identify promising therapeutic targets and develop more effective strategies.

The ubiquitin‐proteasome system (UPS), which is responsible for degrading 80%‐90% of cellular proteins, is involved in multiple biological processes of cancer, including proliferation, metastasis, metabolism and drug resistance.^4^ Moreover, the specificity of human UPS mainly depends on approximately 617 E3 ubiquitin ligases. Zinc finger protein 91 (ZFP91), a novel E3 ubiquitin ligase, is demonstrated to be upregulated in prostate cancer, colon cancer and pancreatic cancer.^5^, ^6^, ^7^ It is reported that ZFP91 facilitates prostate cancer proliferation via NF‐κB pathway and tumorigenesis of colon cancer by upregulating HIF‐1α level.^5^, ^7^ However, the exact role of ZFP91 in pancreatic cancer progression and related signal transduction mechanism remains unclear.

It is proved that epithelial‐to‐mesenchymal transition (EMT) plays a pivotal role in drug resistance.^8^ EMT can be induced by various factors, such as transforming growth factor beta (TGF‐β),^9^ hepatocyte growth factor (HGF)^10^ and platelet‐derived growth factor (PDGF).^11^ β‐catenin is regarded as a key transcriptional coactivator in EMT.^12^ During EMT, β‐catenin translocates into the nucleus from cytoplasm and promotes transcription of genes that induce EMT. In pancreatic cancer, β‐catenin can also accelerate cell proliferation by inducing EMT.^13^ Several E3 ligases are shown to participate in degradation of β‐catenin, such as mind bomb 1, β‐TrCP and Jade1.^14^, ^15^

In our study, we demonstrated that the mRNA expression of ZFP91 gene was significantly upregulated in pancreatic cancer compared with normal pancreas tissues according to Gene Expression Omnibus (GEO). High expression of ZFP91 gene negatively correlated with overall survival in patients with pancreatic cancer. ZFP91 knockdown (KD) suppressed pancreatic cancer cell proliferation in KP4 and HuP‐T3 cell lines. Meanwhile, The ZFP91 knock‐down cells also exhibited attenuated migration and invasion ability in wound healing and transwell invasion assays. The gene set enrichment analysis (GSEA) revealed that several TGF‐β, invasiveness and β‐catenin‐related gene sets were enriched in ZFP91 high‐expressed pancreatic cancer patients. Afterwards, the protein level of active β‐catenin and EMT markers decreased in ZFP91 KD cells. Then, β‐catenin agonist promoted ZFP91 KD KP4 and HuP‐T3 cell proliferation and migration. Furthermore, ZFP91 KD also enhanced the effect of chemotherapies in vivo and in vitro. Our study revealed a new mechanism that ZFP91 promoted pancreatic cancer cell proliferation, migration and invasion through activating β‐catenin pathway, implying that ZFP91 might be a potential therapeutic target for pancreatic cancer.

## 2 MATERIALS AND METHODS

### 2.1 Bioinformatics analysis

The mRNA data were obtained from GEO data set. The survival of PDAC patients was analysed using the log‐rank test. The patients from TCGA data set with the top 5% ZFP91 expression were defined as high ZFP91 group, while those with the lowest 5% ZFP91 expression belonged to low ZFP91 group. Then, GSEA analysis was performed based on the data from above high and low ZFP91 groups of patients to identify main biological pathways regulated by ZFP91.

### 2.2 Clinical samples

The paired tumour and adjacent normal tissues were collected from patients diagnosed with pancreatic cancer undergoing pancreaticoduodenectomy in the Department of Pancreatic Surgery of Nanjing Drum Tower Hospital. Informed consent was signed by all patients. All experimental protocols were approved by the research ethics committee of Nanjing Drum Tower hospital, and research ethics approval for this project was granted from the same institution.

### 2.3 Cell culture and ZFP91 knockdown

Human PDAC cell lines MIA‐PaCa2, CFPAC‐1, HPAC, AsPC‐1, KP4 and HuP‐T3 were cultured in Dulbecco's modified Eagle's medium (DMEM, Invitrogen, CA, United States) supplemented with 10% foetal bovine serum (FBS, Invitrogen). ZFP91 shRNA lentiviruses were obtained from Obio Technology (Shanghai, China). Both KP4 and HuP‐T3 cells were infected with ZFP91 shRNA lentivirus and selected against puromycin (2 μg/mL) for 7 days. Then, the efficiency of ZFP91 knock‐down was examined by western blot analysis. The following target sequences were used in our experiments:

ZFP91‐1: CGGCGCGACTCCTATGCATAGAAACTCGAGTTTCTATGCATAGGAGTCGCGTTTTTG;

ZFP91‐2: CGGCGCTATTTGCAGCACCACATTCTCGAGAATGTGGTGCTGCAAATAGCGTTTTT.

### 2.4 Western Blot analysis

Total proteins from human tissues and cultured cells were extracted using RIPA lysis buffer (Thermo Fisher, USA) containing protease inhibitor cocktail (Roche, Switzerland). After sodium dodecyl sulphate‐polyacrylamide gel (SDS‐PAGE) electrophoresis, the proteins of lysates were transferred to polyvinylidene fluoride membrane and blocked by 5% BSA in TBST for 1 hour at room temperature. Next, the membrane was incubated with primary antibodies against ZFP91 (ab30970, abcam, UK), β‐catenin (9582S, CST, USA), active‐β‐Catenin (8814S, CST, USA), E‐cadherin (14472S, CST, USA), Claudin 1 (ab15098, abcam, UK), Snail (3879S, CST, USA), Slug (9585S, CST, USA), Vimentin (ab92547, abcam, UK) and GAPDH (2118S, CST, USA). Secondary anti‐rabbit and anti‐mouse horseradish Peroxidase (HRP)‐conjugated antibodies were used subsequently. Finally, blots were developed with ECL Western Blotting Substrate (Thermo Fisher, USA). Signals were detected by ChampChemi Top 420 (Sagecreation, China).

### 2.5 Colony formation assay

For colony formation assay, 1000 cells were seeded to each well in 6‐well plates upon the preparation of single‐cell suspensions. Ten days later, the cells were fixed and stained with 0.4% crystal violet dissolved in methanol. The plates were washed thoroughly three times with pure water and then scanned.

### 2.6 Growth curve analysis

For growth curve analysis, cells were seeded in 6‐well plates at a density of 1 × 10^5^ cells per well. After overnight incubation, the culture medium was discarded and replaced with fresh medium. Cells were harvested by 0.25% trypsin and counted with a haemocytometer 0, 24, 48, 72 hour after changing the medium.

### 2.7 Cell viability assays

Cell viability was measured using MTT assay. PDAC cells were seeded in 96 well plates at a density of 4000 cells per well. To prepare FOLFIRINOX treatment, all the drugs were dissolved in Milli‐Q water and mixed, and the final concentration of 3 mg/mL fluorouracil (S1209, Selleck, China), 0.25 mg/mL oxaliplatin (S1224, Selleck, China), 2 mg/mL irinotecan (S1198, Selleck, China) and 2 mg/mL leucovorin (S1236, Selleck, China) is regarded as 100% FOLFIRINOX. After 24 hour, different concentrations of Gemcitabine (S1714, Selleck, China) or FOLFIRINOX were added to the medium to treat the cells. 72 hour later, the supernatant of each well was discarded and MTT agent (500 ug/mL) dissolved in serum‐free DMEM medium was added to each well. The supernatant was then discarded again after 2 hour. Finally, DMSO was added to dissolve the formazan, and the absorbance at 570 nm was measured by Multiscan Spectrum (SpectraMax M5).

### 2.8 Wound healing assay

PDAC cells were cultured in 6‐well plates at a density of 5 × 10^4^ cells/mL, 2 mL/well overnight. After 48 hour, a straight scratch was made with a 20 μL pipette tip in order to simulate a wound. The cell debris was removed, while the edge of the scratch was smoothed by washing with PBS twice. At 0, 24 and 48 hours after the scratch, digital images of cells were acquired under a phase contrast microscope (Nikon ECLIPSE TE2000‐S, Japan). To quantify the closure of the scratch, the difference between the remaining scratch area at time 0, 24 and 48 hours was evaluated. Migration rate was expressed as percentage of scratch closure on an initial area basis.

### 2.9 Cell invasion assay

An in vitro cell invasion assay was performed using 24‐well transwell chambers (8 μm pore size, Corning, USA) with polyvinylpyrrolidone‐free polycarbonate filters coated with Matrigel (Corning, USA). PDAC cells were cultured in the upper chamber with serum‐free medium. Then, the medium supplemented with 10% FBS was added to the lower chamber. After 48 hours, cells that invaded through the membrane to the lower chamber were fixed with methanol, stained with 0.1% crystal violet. Furthermore, the stained crystal violet was also extracted by 33% acetic acid and the absorbance at 570 nm was measured.

### 2.10 Animal experiment

The animal experiments of this study were approved by the Ethics Committee of Nanjing Drum Tower Hospital in accordance with Institutional Animal Care and Use Committee guidelines. Male athymic nude mice (NCr‐nu/nu) were bred in‐house, and 7‐ to 8‐week‐old ones were adopted for implantation. 1 × 10^6^ KP4 scramble or KP4 ZFP91 KD cells were injected orthotopically to the pancreas of each mouse, and 7 days later, those mice were randomized into four treatment groups (n = 7): (a) Control group; (b) shZFP91 group; (c) FOLFORINOX treatment group; and (d) FOLFORINOX treatment plus shZFP91 group. For FOLFIRINOX treatment, each mouse was subjected to tail vein injection with a dosage of 24 mg/kg leucovorin, 2 mg/kg oxaliplatin, 20 mg/kg fluorouracil and 20 mg/kg irinotecan once a week. Therapy was maintained for 4 weeks, and animals were sacrificed by then. Tumour and body weight were measured as well.

### 2.11 Statistical analysis

Data were presented as mean ± SEM from three or more independent experiments. Statistical analysis was carried out by GraphPad Prism 8 software (San Diego, USA). Differences among groups were evaluated by the Student's *t* test or one‐way analysis of variance (ANOVA). *P*‐values were calculated, and statistical significance is displayed as ^*^ *P* <.05, ^**^ *P* <.01. ^***^ *P* <.001, ^****^ *P* <.0001, NS: not significant.

## 3 RESULTS

### 3.1 ZFP91 overexpression is prevalent in pancreatic cancer and negatively correlated with overall survival

**FIGURE 1:**
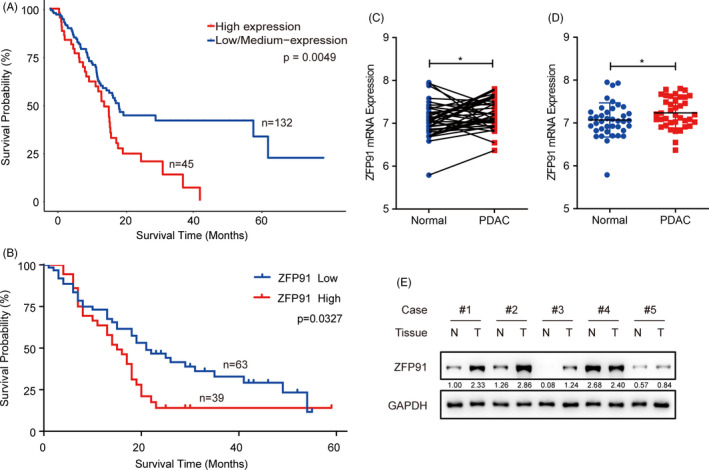
Frequent upregulation of ZFP91 in pancreatic cancer. A and B, Kaplan‐Meier survival analysis showed pancreatic cancer patients with copy number alterations in ZFP91 had worse survival than those without alterations from TCGA and GEO data sets. C and D, ZFP91 mRNA expression was upregulated in pancreatic tumour compared with paired and unpaired adjacent normal tissues in GEO data set. E, Expression of ZEP91 in pancreatic cancer (T, n = 5) and adjacent normal tissues (N, n = 5) was determined by Western blot analysis. ^*^ *P* <.05

The correlation between ZFP91 expression and the clinical outcome in TCGA and GEO data sets was analysed. The Kaplan‐Meier analysis showed that high expression in ZFP91 gene was significantly associated with worse survival in the patients (Figure 1A,B). According to GEO data set, ZFP91 expression was also remarkably increased in tumour tissues compared with adjacent normal tissues (Figure 1C,D). Then, we detected the protein level of ZFP91 in five human samples and ZFP91 expression was elevated in most tumour tissues compared with adjacent normal tissues (Figure 1E).

### 3.2 ZFP91 KD attenuates the proliferation of pancreatic cancer cells

**FIGURE 2:**
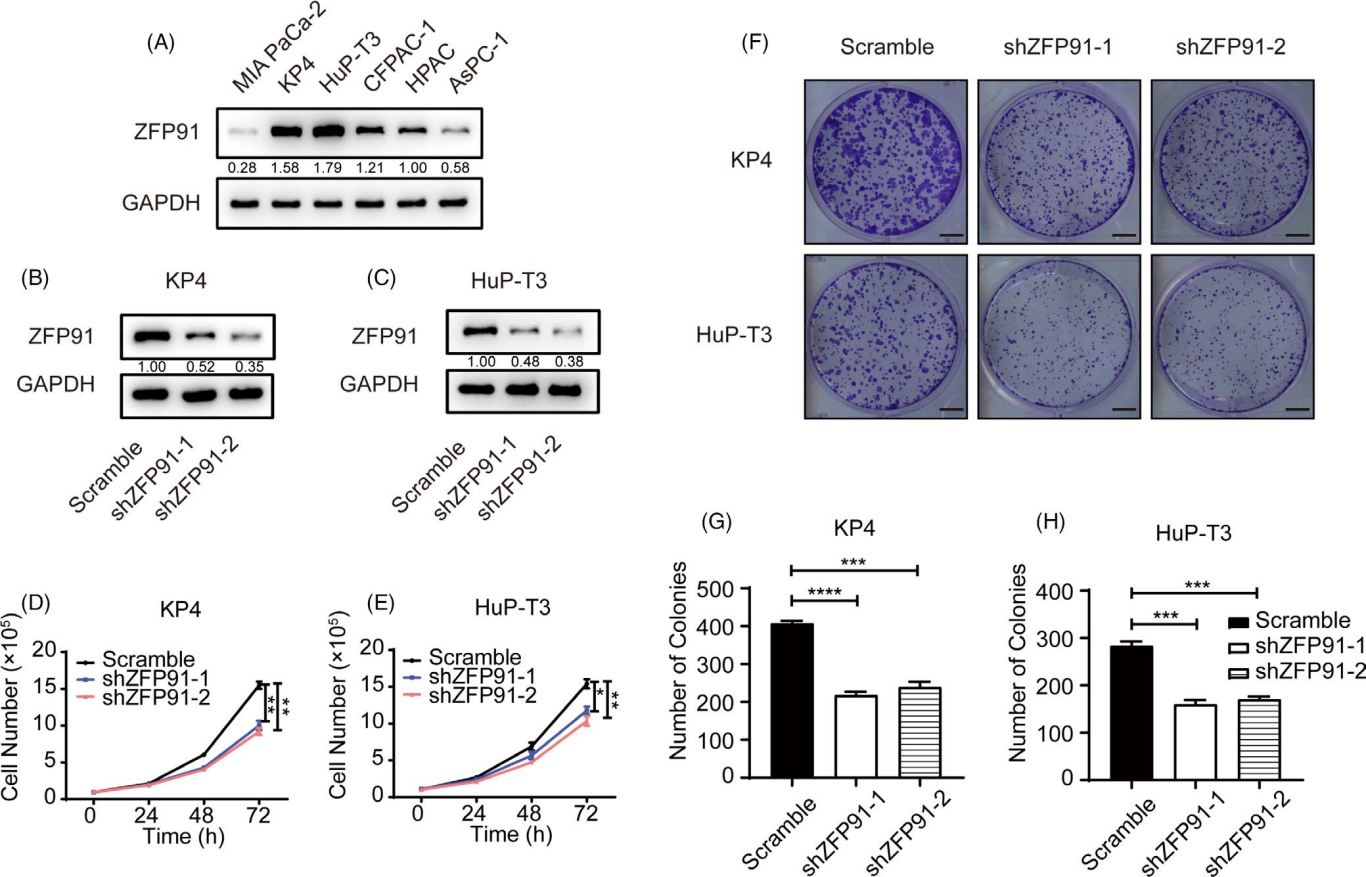
ZFP91 KD attenuates pancreatic cancer cell proliferation. A, Western blot analysis of ZFP91 expression in six human pancreatic cancer cell lines. B and C, Western blot analysis of ZFP91 KD efficiency in KP4 and HuP‐T3 cells. D and E, Growth curve of scramble and ZFP91 KD cells. F‐H, Colony formation of scramble and ZFP91 KD cells. Scale bar: 5 mm. ^*^ *P* <.05, ^**^ *P* <.01, ^***^ *P* <.001, ^****^ *P* <.0001

KP4 and HuP‐T3 has relatively high expression of ZFP91 compared with other pancreatic cancer cell lines (Figure 2A). Lentivirus was used to knock down ZFP91 in KP4 and HuP‐T3 cells (Figure 2B,C). According to growth curve analysis, ZFP91 KD impaired proliferation ability in pancreatic cancer cells (Figure 2D,E). Colony formation assay also showed a significantly lower colony efficiency after ZFP91 KD (Figure 2F‐H). These results suggested that deficiency of ZFP91 expression contributed to decreased proliferation in pancreatic cancer.

### 3.3 ZFP91 KD impairs migration and invasion ability of pancreatic cancer cells

**FIGURE 3:**
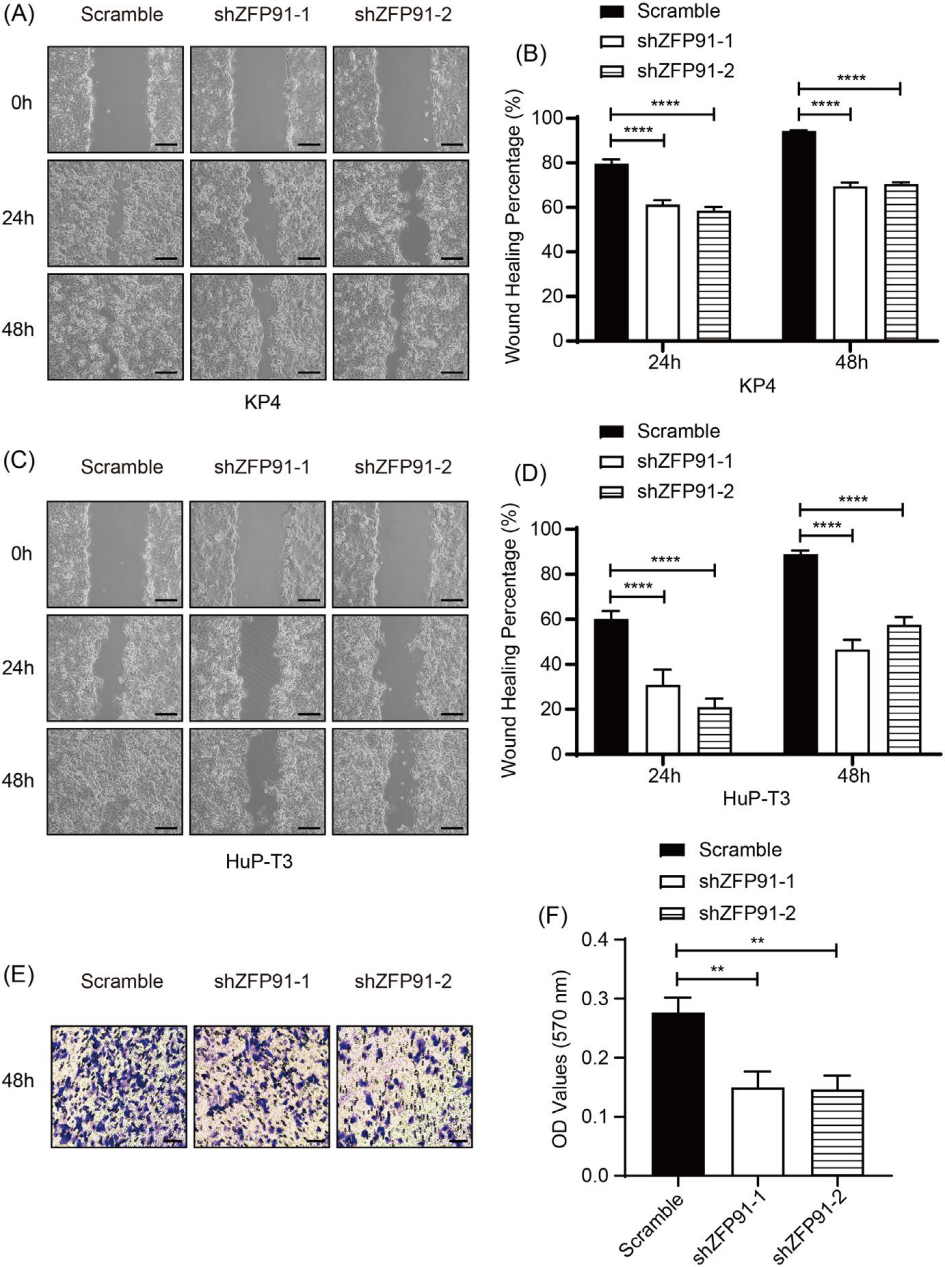
ZFP91 KD inhibits pancreatic cancer cell migration and invasion in vitro. A‐D, Cell migration was determined by wound healing assay in KP4 scramble and ZFP91 KD cells, as well as in HuP‐T3 cells. Scale bar: 200 μm. E, Cell invasion was determined by transwell invasion assay in KP4 scramble and ZFP91 KD cells at 48  h after cell seeding, and (F) the absorbance at 570 nm of the stained crystal violet dissolved in 33% acetic acid was measured. Scale bar: 100 μm. **P <.01, ***P <.001, ****P <.0001

Wound healing assay was carried out in both KP4 and HuP‐T3 cells to evaluate the migration rate of pancreatic cancer in vitro. Compared with scramble cells, ZFP91 KD cells showed delayed wound healing process (Figure 3A‐D). In transwell invasion assay, ZFP91 KD KP4 cells also exhibited impaired invasion ability (Figure 3E,F), revealing that ZFP91 was involved in migration and invasion of pancreatic cancer cells.

### 3.4 GSEA analysis reveals EMT related gene sets are associated with ZFP91 level

**FIGURE 4:**
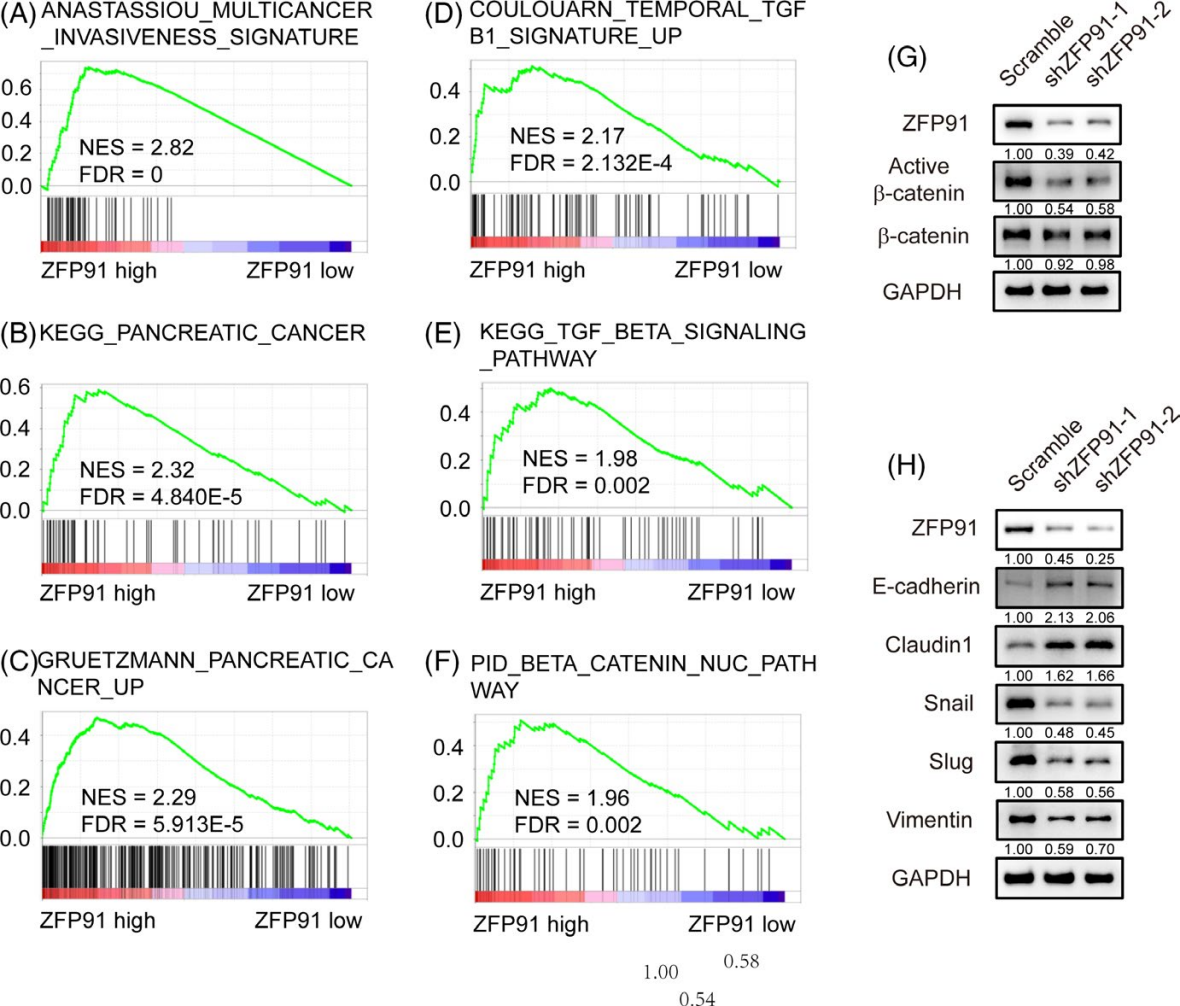
β‐catenin is a downstream gene of ZFP91. A‐F, GSEA analysis was conducted to show cancer invasiveness, pancreatic cancer, TGF‐β signalling and β‐catenin related gene sets related to ZFP91 expression. G, Expression of β‐catenin, active β‐catenin in KP4 scramble and ZFP91 KD cells was determined by Western blot analysis, as well as (H) epithelial‐to‐mesenchymal transition (EMT) markers

To elucidate the mechanisms by which ZFP91 promoted aggressive behaviour in pancreatic cancer, GSEA analysis was performed, suggesting that ZFP91 level was associated with multicancer invasiveness and pancreatic cancer (Figure 4A‐C). TGF‐β related gene sets were also enriched in patients with high ZFP91 levels (Figure 4D,E). Furthermore, β‐catenin‐related gene sets were altered significantly based on different expression of ZFP91 (Figure 4F). To further confirm the relationship between ZFP91 and β‐catenin, we examined β‐catenin expression in KP4 ZFP91 KD cells. The Western blot analysis showed that the expression of active β‐catenin was significantly downregulated in KP4 ZFP91 KD cells than in scramble cells (Figure 4G). Since β‐catenin was reported to play a key role in EMT,^13^ we also detected EMT markers in ZFP91 KD cells and noticed increased expression of the epithelial markers such as E‐cadherin and Claudin 1, as well as decreased expression of Snail, Slug and Vimentin, which were defined as the mesenchymal markers (Figure 4H). Collectively, the above results indicated that β‐catenin might play an important role in the development of pancreatic cancer regulated by ZFP91.

### 3.5 Activation of β‐catenin pathway reverses the phenotypes in cell proliferation and migration induced by ZFP91 KD

**FIGURE 5:**
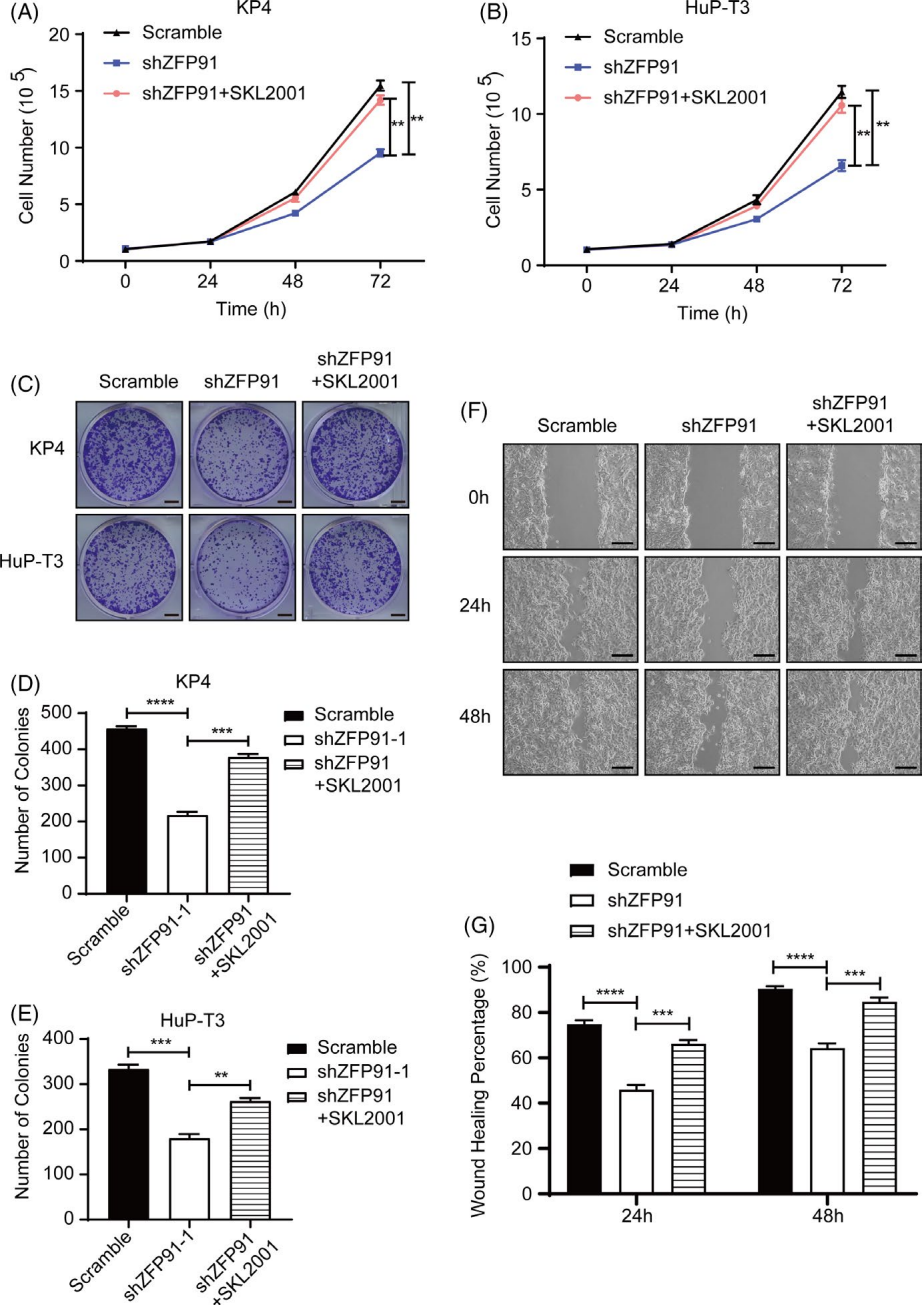
ZFP91 regulates pancreatic cancer cell proliferation and migration through β‐catenin pathway. KP4 scramble and ZFP91KD cells were pretreated with DMSO or 30uM SKL2001 for 24 h, and then cell proliferation was determined by (A, B) growth curve and (C‐E) colony formation assay, as well as in HuP‐T3 cells. Scale bar: 5 mm. F and G, Cell migration was determined by wound healing assay in pretreated KP4 cells. Scale bar: 200 μm. **P <.01, ***P <.001, ****P <.0001

To further confirm the relationship between ZFP91 and β‐catenin, KP4 and HuP‐T3 KD cells were treated with 30uM SKL2001, a well‐known β‐catenin agonist.^16^ Growth curve analysis showed that SKL2001 significantly accelerated proliferation of KP4 and HuP‐T3 ZFP91 KD cells (Figure 5A,B), as well as colony formation assay (Figure 5C‐E). In addition, the migration rate in KP4 cells interfered with deficiency of ZFP91 was also improved by SKL2001 through the activation of β‐catenin pathway (Figure 5F,G). These findings demonstrated that ZFP91 contributed to tumour progression of pancreatic cancer in a β‐catenin–dependent manner.

### 3.6 ZFP91 KD sensitizes pancreatic cancer to chemotherapies

**FIGURE 6:**
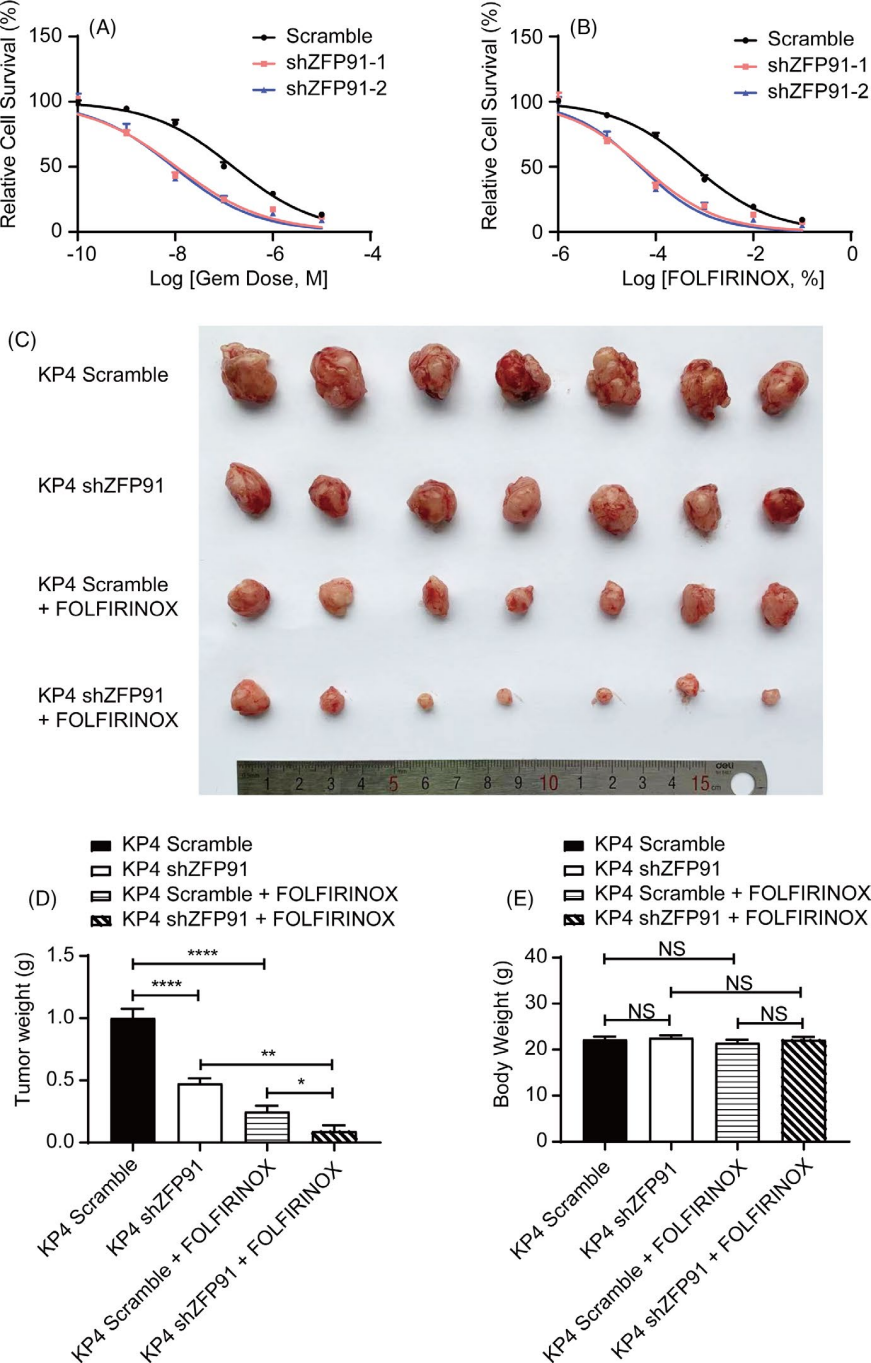
ZFP91 confers chemoresistance in pancreatic cancer in vitro and in vivo. Dose‐response curves for (A) gemcitabine (GEM) and (B) FOLFIRINOX treatment in KP4 scramble and ZFP91 KD cells. KP4 scramble and ZFP91 KD cells were injected orthotopically in athymic‐nude mice. Seven days later, mice were separately subjected to treatment with DMSO or FOLFIRINOX (n = 7) for 4 weeks. C, Representative tumour images upon necropsy were given. D, Tumour weight upon necropsy and (E) body weight before sacrificed were measured. NS, not significant, **P <.01, ****P <.0001

To evaluate whether ZFP91 promotes resistance to chemotherapies, we treated KP4 ZFP91 KD cells with Gemcitabine or FOLFIRINOX in a range of concentrations for 72 hours. The responses to chemotherapies were subsequently determined by MTT assays, which proved that ZFP91 KD cells were more sensitive to both drugs (Figure 6A,B). Furthermore, athymic nude mice were implanted orthotopically with KP4 scramble and ZFP91 KD cells, followed by FOLFIRINOX therapy. KP4 ZFP91 KD cells exhibited a dramatic decrease in tumour volume and weight (Figure 6C,D). Without significantly difference in mouse weight (Figure 6E), the results revealed that the sensitivity of pancreatic cancer to chemotherapies was enhanced by the disruption of ZFP91.

## 4 DISCUSSION

Previous studies have demonstrated that ZFP91 is a tumour promotor in several cancers and elevated in pancreatic cancer.^5^, ^6^, ^7^ In our study, we analysed TCGA and GEO data sets and found that high expression of ZFP91 in pancreatic cancer is associated with worse survival. In addition, higher ZFP91 mRNA and protein expression was detected in pancreatic tumour tissues compared with normal adjacent tissues. Then, we found that ZFP91 KD inhibited the proliferation, migration and invasion of pancreatic cancer cells.

To seek for the downstream of ZFP91, GSEA analysis was performed. Among the gene sets which were associated with ZFP91 level, cancer invasiveness, pancreatic cancer, TGF‐β signalling and β‐catenin related gene sets were found significant. Since EMT was associated with all the three gene sets, we hypothesized that ZFP91 promoted EMT in pancreatic cancer by activating β‐catenin, which was confirmed by western blot analysis. Notably, the inhibitory effect of ZFP91 KD on tumour biological behaviour in KP4 and HuP‐T3 cells was abrogated by the application of β‐catenin agonist SKL2001.

Given that chemoresistance posed a great threat to long‐term survival of patients with pancreatic cancer,^17^, ^18^ we tried to find out the correlation between ZFP91 and chemoresistance. According to the in vitro drug response and mouse model, we concluded that disruption of ZFP91 improved chemosensitivity in pancreatic cancer.

However, the exact molecular interaction between ZFP91 and β‐catenin has not been clarified in this study. It was reported that MTH9 could blocked GSK3β ubiquitination so as to upregulate β‐catenin level and finally drive aggressive behaviour in hepatocellular carcinoma.^19^ TRAF6, an E3 ligase, the same as ZFP91, was proved to mediate GSK3β ubiquitination and regulate inflammatory response.^20^ Thus, we assumed that ZFP91 might contribute to ubiquitination and degradation of GSK3β, which was responsible for phosphorylation and inactivation of β‐catenin. Nonetheless, the truly underlying mechanism warranted further investigation.

Taken together, our study clearly demonstrated that ZFP91 promoted pancreatic cancer proliferation, migration and invasion, which was mediated by β‐catenin signalling. ZFP91 also facilitated EMT and chemoresistance in pancreatic cancer. Our results implicated that combination of ZFP91 and β‐catenin inhibition might be a novelty and effective treatment approach to pancreatic cancer. Moreover, chemotherapy enhanced by ZFP91 inhibition is also a good choice.

## CONFLICT OF INTEREST

The authors have declared no conflict of interest.

## Data Availability

The data that support the findings of this study are available from the corresponding author upon reasonable request. The data are not publicly available due to piracy or ethical restrictions.
